# Dexmedetomidine alleviates pulmonary edema through the epithelial sodium channel (ENaC) via the PI3K/Akt/Nedd4-2 pathway in LPS-induced acute lung injury

**DOI:** 10.1007/s12026-021-09176-6

**Published:** 2021-02-28

**Authors:** Yuanxu Jiang, Mingzhu Xia, Jing Xu, Qiang Huang, Zhongliang Dai, Xueping Zhang

**Affiliations:** 1 Department of Anesthesiology, Shenzhen People’s Hospital (The Second Clinical Medical College, Jinan University, The Fist Affiliated Hospital, Southern University of Science and Technology), Shenzhen, 518020 China; 2 Shenzhen Anesthesiology Engineering Center, Shenzhen, 518020 China; 3 Department of Pathology, Shenzhen People’s Hospital (The Second Clinical Medical College, Jinan University, The Fist Affiliated Hospital, Southern University of Science and Technology), Shenzhen, 518020 China; 4 Hubei Community Health Service Center, Luohu Hospital Group, Luohu People’s Hospital, Shenzhen, 518020 China

**Keywords:** Pulmonary edema, Dexmedetomidine, ENaC, AFC, PI3K/Akt/Nedd4-2 signaling pathway

## Abstract

Dexmedetomidine (Dex), a highly selective α_2_-adrenergic receptor (α_2_AR) agonist, has an anti-inflammatory property and can alleviate pulmonary edema in lipopolysaccharide (LPS)-induced acute lung injury (ALI), but the mechanism is still unclear. In this study, we attempted to investigate the effect of Dex on alveolar epithelial sodium channel (ENaC) in the modulation of alveolar fluid clearance (AFC) and the underlying mechanism. Lipopolysaccharide (LPS) was used to induce acute lung injury (ALI) in rats and alveolar epithelial cell injury in A549 cells. In vivo, Dex markedly reduced pulmonary edema induced by LPS through promoting AFC, prevented LPS-induced downregulation of α-, β-, and γ-ENaC expression, attenuated inflammatory cell infiltration in lung tissue, reduced the concentrations of TNF-α, IL-1β, and IL-6, and increased concentrations of IL-10 in bronchoalveolar lavage fluid (BALF). In A549 cells stimulated with LPS, Dex attenuated LPS-mediated cell injury and the downregulation of α-, β-, and γ-ENaC expression. However, all of these effects were blocked by the PI3K inhibitor LY294002, suggesting that the protective role of Dex is PI3K-dependent. Additionally, Dex increased the expression of phosphorylated Akt and reduced the expression of Nedd4-2, while LY294002 reversed the effect of Dex in vivo and in vitro. Furthermore, insulin-like growth factor (IGF)-1, a PI3K agonists, promoted the expression of phosphorylated Akt and reduced the expression of Nedd4-2 in LPS-stimulated A549 cells, indicating that Dex worked through PI3K, and Akt and Nedd4-2 are downstream of PI3K. In conclusion, Dex alleviates pulmonary edema by suppressing inflammatory response in LPS-induced ALI, and the mechanism is partly related to the upregulation of ENaC expression via the PI3K/Akt/Nedd4-2 signaling pathway.

## Introduction

Acute lung injury (ALI) is characterized by acute, diffuse, and inflammatory lung injury. Clinically, ALI manifests as severe respiratory distress and intractable hypoxemia. At present, lung protective ventilation support is beneficial to ALI patients [1, 2], but there is no effective drug therapy. Despite the efforts made to cure ALI, its clinical mortality has remained high in recent decades [3]. Pulmonary edema is the central link to the pathogenesis of ALI, which is associated with alveolar epithelial injury and impaired alveolar fluid clearance (AFC) [4]. Studies have shown that maximum AFC results in lower mortality and requires less mechanical ventilation time [5]. Therefore, the timely and effective removal of excess liquid from alveoli is a key goal in the treatment of ALI.

Alveolar fluid clearance is associated with ENaC [6, 7]. Sodium ions (Na^+^) are actively transported into cells through ENaC, and then Na^+^ is pumped into the interstitium through the action of Na, K-ATPase, which leads to an osmotic gradient that drives the transfer of water into the interstitium and into the blood circulation through aquaporin (AQP) [8]. Current research suggests that pulmonary edema may be attributed to the inflammatory response during ALI. Several inflammatory cytokines, including TNF-α and IL-1β, may affect ENaC expression [9–11]. Many studies have indicated that inhibiting the release of inflammatory cytokines may promote AFC by increasing the expression of ENaC[12]. Therefore, inhibition of the inflammatory response may be beneficial for upregulating ENaC expression and reducing pulmonary edema.

Dexmedetomidine (Dex), a highly selective α_2_-adrenergic receptor (α_2_AR) agonist, can reduce pulmonary edema through inhibiting the production of inflammatory cytokines, including TNF-α, IL-1β, and IL-6 in an ALI model [13–16]. Consistent with these studies, our previous studies showed that Dex can attenuate pulmonary edema in LPS-induced ALI, improve PaO_2_, and reduce the inflammatory response. Further research has revealed that Dex increases the expression of aquaporin1 (AQP1) and aquaporin5 (AQP5) in lung tissue [17], indicating that the ability of Dex to reduce pulmonary edema may be related to the promotion of AFC. As 90% of water transport resistance comes from ENaC-mediated Na^+^ transport, it is reasonable to speculate that Dex may alleviate pulmonary edema by stimulating AFC through the upregulation of ENaC expression.

Previous studies have shown that continuous stimulation of the PI3K/Akt signaling pathway promotes Na^+^ absorption in epithelial cells [18]. Other studies have found that insulin and RvD1 can activate the PI3K/Akt signaling pathway, upregulate the expression of ENaC, and promote AFC [19, 20]. Furthermore, Akt increases ENaC activity by inducing Nedd4-2 expression, thereby increasing Na^+^ absorption [21]. Recent studies have found that Dex reduces LPS-induced ALI by activating the PI3K/Akt signaling pathway [15]. These results prompted us to hypothesize that the effect of Dex on ENaC may be related to the PI3K/Akt/Nedd4-2 signaling pathway.

This study was designed to investigate whether Dex can reduce pulmonary edema by promoting AFC in LPS-induced ALI in rats. Additionally, we studied the effect of Dex on ENaC expression and the role of PI3K/Akt/Nedd4-2 signaling in these effects.

## Materials and methods

### Animals

The Medical Faculty Ethics Committee of Shenzhen People’s Hospital, Shenzhen, China, approved all the animal procedure and care protocols. The animal experiments complied with the Guidelines for the Care and Use of Laboratory Animals from the NIH. SPF grade male Wistar rats (6 weeks old, weighing 180~220 g) were purchased from Guangdong Medical Animal Experiment Center (Guangzhou, China). Animal handling was conducted according to the requirements of the animal protection committee of the Second Clinical Medical College of Jinan University (Shenzhen, China). All animals were housed in an air-conditioned room under a 12-hour dark/light cycle and were given free access to water and food.

### Experimental protocols

The rats were anesthetized with sodium pentobarbital (50 mg/kg, i.p. injection). The skin of the left thigh was disinfected with ethanol, the skin was cut open, the femoral vein was exposed, a 24^#^ trocar was inserted and fixed, and then 20 mg/kg LPS (*Escherichia coli* 055:B5) was injected (over 10 min) to induce ALI. After LPS or saline injection, the neck skin was disinfected, the trachea was exposed, and a homemade tracheal catheter was inserted. Eight hours after LPS administration, all rats were sacrificed. The study consisted of two parts. The first part was the survival study, in which 10 animals were used in each group. The survival rate of the rats was recorded every 2 h for 3 days after LPS administration, and the dosage of Dex was determined.

In the second part, the rats were divided into four groups (*n* = 6 per group): the control group, LPS group, LPS+Dex group, and LPS+Dex+LY294002 group. The rats in the control group were intravenously administered 0.9% normal saline (5 ml/kg). The rats in the LPS group were intravenously administered 20 mg/kg LPS. The rats in the LPS+Dex group were intravenously administered 20 mg/kg LPS and then intraperitoneally administered 100 μg/kg Dex (St. Louis, MO, USA). The rats in the LPS+Dex+LY294002 group were intravenously administered 3 mg/kg LY294002 (Monmouth Junction, NJ, USA) 30 min prior to LPS injection and then administered 100 μg/kg Dex.

### Histopathological studies

The lower lobe of the right lung was collected, fixed with 10% neutral formaldehyde for 24 h, embedded in paraffin, and stained with H&E for light microscopy analysis. Histological lung injury was scored based on alveolar edema, pulmonary capillary congestion, neutrophil infiltration, and the thickness of the alveolar septum in five random fields in a blinded manner using light microscopy. Lung sections were scored as 1 (no or very slight pathological changes), 2 (slight pathological changes), 3 (moderate pathological changes), or 4 (severe pathological changes). Evaluation scores were added to the total injury score.

### Measurement of cytokines

After the rats were sacrificed, the main bronchus was exposed. The right bronchus was ligated, and a homemade tracheal catheter was inserted into the main bronchus. Then, 2 ml cold phosphate-buffered saline (PBS) was infused into the left lung and extracted three times. The bronchoalveolar lavage fluid (BALF) was centrifuged at 1200 × g for 10 min at 4 °C. The supernatant was separated into aliquots and stored at − 70°C. An aliquot of BALF supernatant was used to assay the levels of TNF-α, IL-1β, IL-6, and IL-10 by the ELISA kit (Jianglai Biotechnology , Shanghai, China) according to the manufacturer’s instructions.

### MPO activity assay in lung tissues

The middle lobe of the right lung was removed immediately after the animals were exsanguinated. Then, the activity of MPO in the lung tissue was determined by an MPO detection kit (Jianglai Biotechnology, Shanghai, China) according to the manufacturer’s instructions.

### Arterial oxygen tension (PaO_2_) assay

Arterial blood (0.5 ml) was extracted from the right common carotid artery before the rats were sacrificed for blood gas analysis, and PaO_2_ was measured with a blood gas analyzer.

### Measurement of the lung wet/dry weight ratio

At the end of the experiments, we immediately removed the upper lobe of the right lung and precisely measured the lung wet weight. After that, we placed the lung tissues in a constant temperature oven at 75 °C for 24 h and measured the lung dry weight. Finally, the W/D (wet/dry) ratio was calculated to evaluate the extent of pulmonary edema.

### Measurement of alveolar fluid clearance

The AFC (alveolar fluid clearance) was determined by measuring the Evans blue-labeled albumin concentration. First, 5% bovine serum albumin perfusion solution labeled with Evans blue (St. Louis, MO, USA) was injected (5 ml/kg) into the left lung via the trachea, and 2 ml oxygen was injected to facilitate distribution. The rats were ventilated with 100% oxygen, and the positive end expiratory pressure was kept at 2~3 cm H_2_O during the baseline period to maintain lung tension. These tissue units were wrapped with plastic wrap and then incubated in a 37 °C water bath for 1 h. The alveolar fluid was immediately aspirated, and labeled albumin was measured by a spectrophotometer at 620 nm. AFC was calculated based on the following formula: AFC (%) = [(*C*_f_ − *C*_i_)/C_f_] × 100%, where *C*_i_ represents the concentration of injected Evans blue-labeled 5% albumin and *C*_f_ represents the final concentration of Evans blue-labeled 5% albumin.

### Cell culture and treatment

A549 cells (Cell Bank of the Chinese Academy of Sciences , Shanghai, China) were seeded in culture dishes at a density of 1 × 10^6^ cells/cm^2^ and cultured in a 5% CO_2_ and 95% air atmosphere in Dulbecco’s modified Eagle medium (DMEM) with 10% fetal bovine serum, 0.1 mg/ml streptomycin, and 100 U/ml penicillin. The culture medium was changed every 2 days. For all experiments, the cells were subcultured in six-well plates. Once the cells reached 80% confluence, they were serum-starved for 24 h. Following starvation, the cells were treated with LPS (1 μg/ml) in the presence or absence of Dex (10 μΜ). IGF-1 (Sigma-Aldrich, USA) was used at a concentration of (200 ng/ml) 1 h prior to LPS (1 μg/ml) administration to activate PI3K; LY294002 was used at a concentration of (10 μΜ) 30 min prior to LPS (1 μg/ml) administration to inhibit PI3K.

### Cell viability assay

The CCK8 assay was performed to measure cell viability. The cells (100 μl/well) were cultured in a 96-well plate for 24 h. Then, the cells were incubated with 1 μg/ml LPS for 12 h in the absence or presence of Dex. Afterwards, 10 μl CCK8 (St. Louis, MO, USA) solution was added to each well, the culture plate was incubated in the incubator for 2 h under 5% CO2 at 37°C, and the absorbance at 490 nm was measured with a microplate reader.

### LDH activity assay

The cells (100 μl/well) were cultured in a 96-well plate for 24 h. Then, they were incubated with 1 μg/ml LPS for 12 h in the absence or presence of Dex. The supernatant was collected to measure lactate dehydrogenase (LDH) activity by using an LDH assay kit (Cambridge, UK) according to the manufacturer’s instructions. The absorbance at 490 nm was measured with a microplate reader.

### Immunohistochemical analyses

Lung tissues were fixed in 10℅ neutral formaldehyde solution, and paraffin tissue sections were produced. The paraffin sections were then baked overnight in a 60°C oven, dewaxed with dimethyl benzene, dehydrated in gradient ethanol solutions, repaired with 500 ml EDTA antigen repair solution, treated with 50 μl 3% hydrogen peroxide solution at room temperature for 20 min to block endogenous peroxidase activity, and rinsed with TBS 3 times (3 min each time). Then, 5% normal goat serum solution was added at room temperature for 20 min, and the superfluous liquid was discarded without washing. Diluted primary antibody was added, and the tissues were incubated at room temperature for 30 min and washed with TBS 3 times (3 min each time). Secondary antibody (biotinylated goat anti-rabbit IgG) was added, and the tissues were incubated at room temperature for 30 min and washed with TBS 3 times (3 min each time). After that, the cells were stained with 3,3’-diaminobenzidine for 3~5 min. PBS was used instead of primary antibody for the negative control group. The average optical densities (AODs) of α-, β-, γ-ENaC were measured by an imaging analysis system.

### Western blot analysis of rat lung tissues and A549 cells

Proteins were obtained with RIPA lysis buffer (50 mM Tris (pH 7.4), 150 mM NaCl, 1% Triton X-100, 1% sodium deoxycholate, 0.1% SDS, sodium orthovanadate, sodium fluoride, EDTA, and leupeptin) and PMSF. The protein concentrations of the supernatants were determined by using a BCA protein assay kit. The proteins were separated by sodium dodecyl sulfate-polyacrylamide gel electrophoresis (SDS-PAGE) and transferred to PVDF membranes. Tris buffer solution containing 5% skim milk powder was used to block the membrane for 1 h at room temperature. The membrane was permeabilized with PBS containing 0.05% Tween 20 and washed with PBST 5 times (5 min each time). Then, the membrane was incubated overnight at 4°C with the following primary antibodies: α-, β-, and γ-ENaC (Abcam, Cambridge, UK), Akt, p-Akt, Nedd4-2, and β-actin (CST, Boston, MA, USA). The membrane was incubated with a horseradish peroxidase (HRP)-labeled goat anti-rabbit antibody (Abcam, Cambridge, UK) at room temperature for 3 h, and the membrane was washed with PBST 5 times (5 min each time). Finally, the bands were visualized using an enhanced chemiluminescence kit (ECL) with a UVP gel imaging system (Upland, CA, USA). The band intensities were analyzed with ImageJ software

### Statistical analysis

All data are expressed as the mean ± standard deviation (SD). The significance of the differences among the four groups was tested using one-way ANOVA followed by a least significant difference (LSD) multiple comparison test. Survival analysis was performed using the Kaplan-Meier method, and comparisons between groups were made using the log rank test. *p* values of < 0.05 were considered statistically significant.

## Results

### Dexmedetomidine increased the survival rate of rats with LPS-induced ALI

**Fig. 1:**
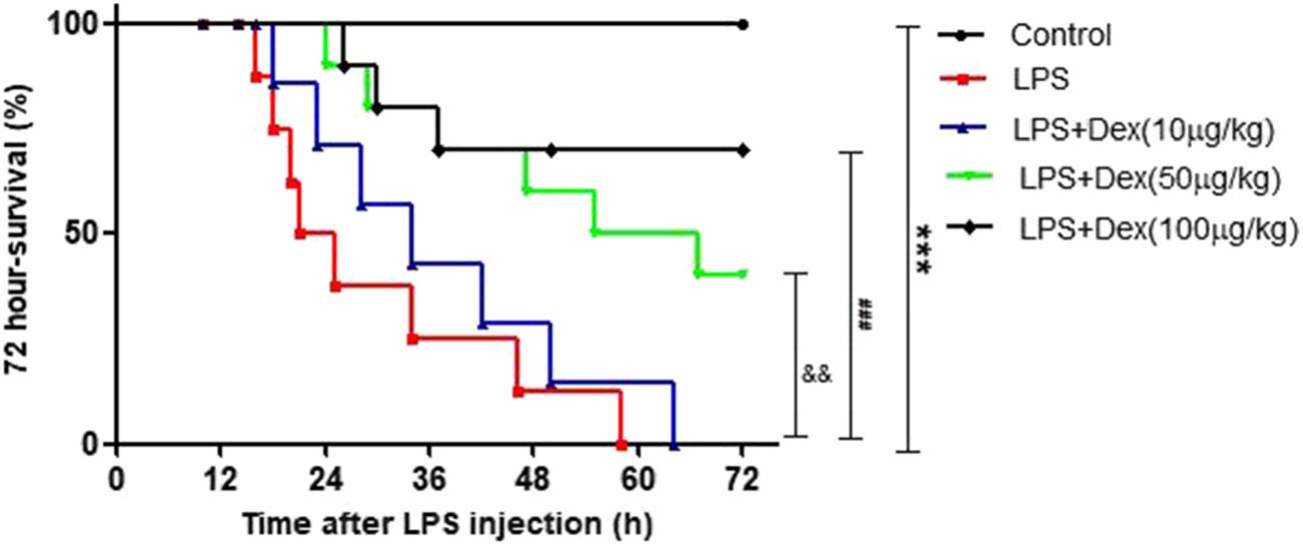
Effect of Dex on the survival rate of rats with LPS-induced ALI. The rats were immediately intraperitoneally injected with Dex (10, 50, or 100 μg/kg) after intravenous injection of LPS (20 mg/kg). Survival was monitored for 72 h. The data are presented as the means ± SD. ***P < 0.001 vs the control group; ^###^*P* < 0.001 vs the LPS group, ^&&^*P* < 0.01 vs the LPS group

To determine the therapeutic effect of Dex on ALI, we observed the effect of Dex on the survival rate of rats with LPS-induced ALI. As shown in Fig. 1, Dex had a dose-dependent effect on the survival rate of rats with ALI, and 100 μg/kg Dex had the best therapeutic effect. Therefore, 100 μg/kg Dex was used in vivo.

### Dexmedetomidine alleviated LPS-induced ALI

**Fig. 2:**
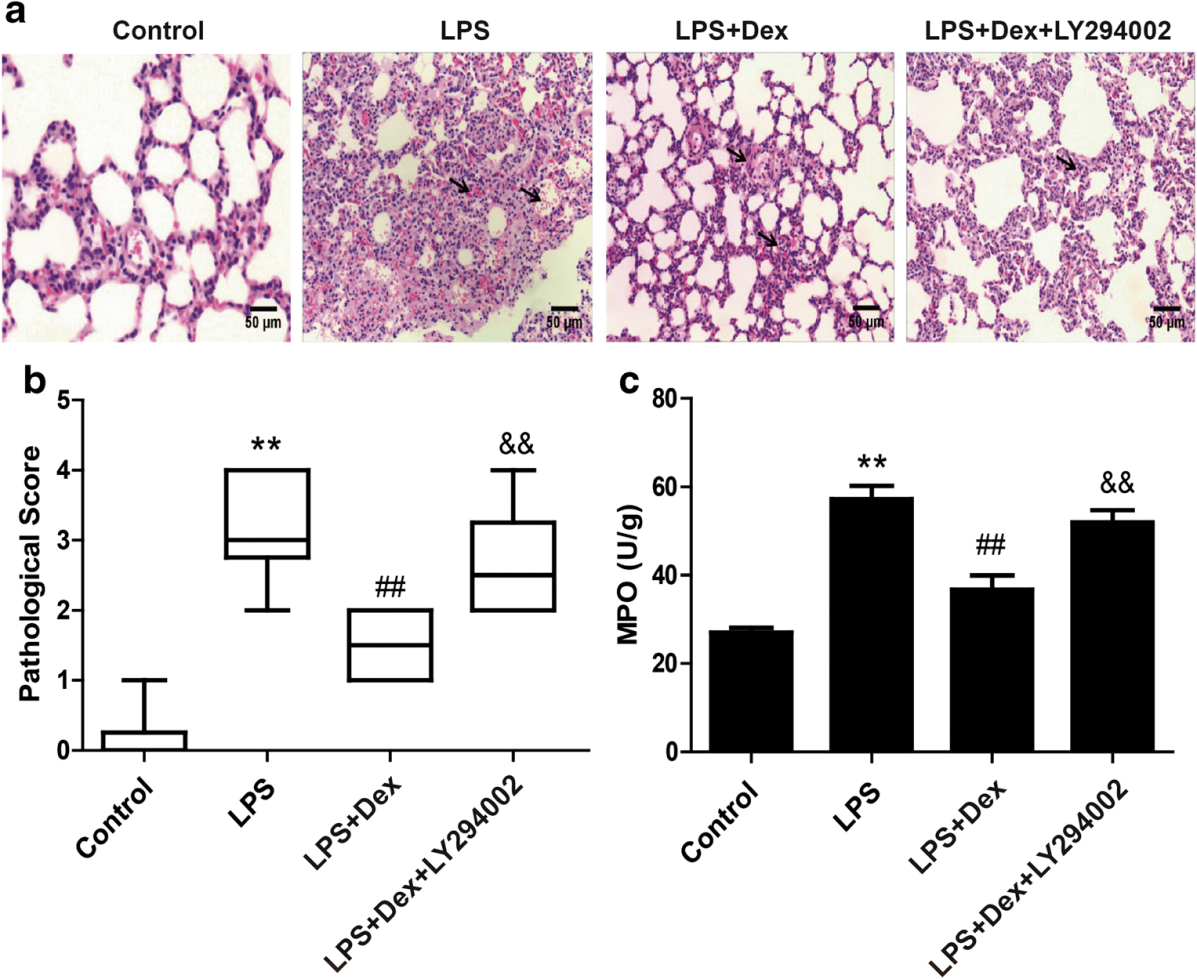
Effect of Dex on LPS-induced ALI. Rats with LPS (20 mg/kg)-induced ALI were treated with Dex (100 μg/kg). To investigate whether the protective effect of Dex is PI3K-dependent, LY294002 (3 mg/kg), a PI3K inhibitor, was given 30 min prior to LPS administration. Eight hours later, the rats were sacrificed by bloodletting. **a** Lung tissues were assessed by histopathology (H&E staining, magnification, ×200). **b** Lung injury scores. **c** MPO level. The data are presented as the means ± SD. **P < 0.01 vs the control group; ^##^*P* < 0.01 vs the LPS group; ^&&^*P* < 0.01 vs the LPS+Dex group

To evaluate whether Dex can alleviate LPS-induced ALI, we first evaluated pulmonary histological alterations by H&E staining. In the control group, the lung structure was intact, and the alveolar cavity was clear and free from inflammatory cell infiltration. Compared with control treatment, LPS induced significant changes in lung injury, namely, interstitial edema, alveolar septum thickening, and a large amount of inflammatory cell infiltration, as evidenced by an increase in the lung injury score. Dex treatment significantly alleviated the pathological alterations induced by LPS (Fig. 2a). Subsequently, we measured lung injury scores and MPO levels, and the results showed that the LPS group had the highest lung injury score and MPO levels. Compared to control treatment, Dex treatment reduced lung injury scores (Fig. 2b) and MPO levels (Fig. 2c). However, the Dex-induced protection against lung injury was blocked by treatment with LY294002, suggesting that Dex plays a protective role against LPS-induced ALI in a PI3K-dependent manner.

### Dexmedetomidine reduced the inflammatory response in LPS-induced ALI

**Fig. 3:**
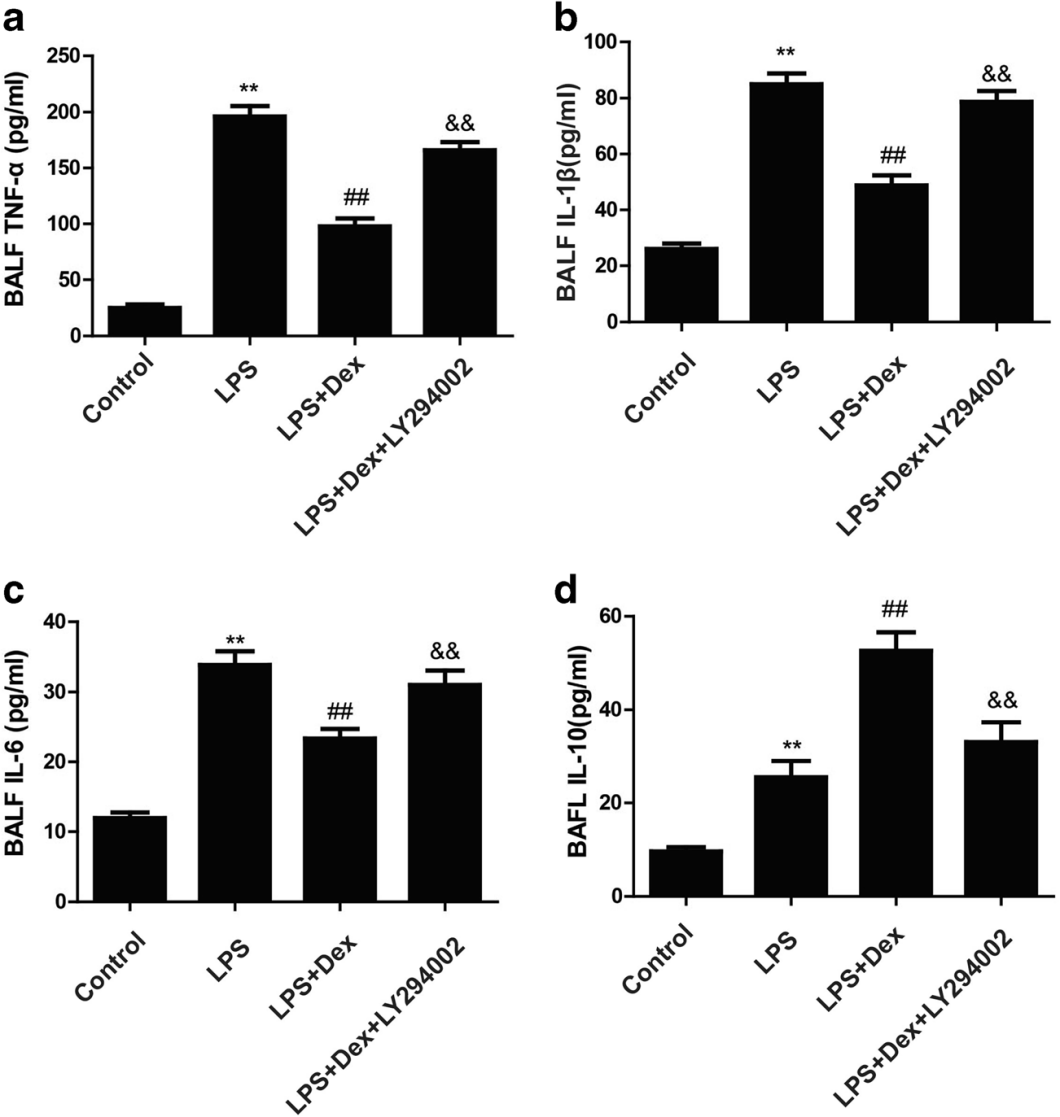
Effect of Dex on the inflammatory response in LPS-induced ALI. Rats with LPS (20 mg/kg)-induced ALI were treated with Dex (100 μg/kg). To investigate whether the protective effect of Dex is PI3K-dependent, LY294002 (3 mg/kg), a PI3K inhibitor, was given 30 min prior to LPS administration. Eight hours later, the rats were sacrificed by bloodletting. Bronchoalveolar lavage fluid was collected to determine the concentrations of TNF-α (**a**), IL-1β (**b**), IL-6 (**c**), and IL-10 (**d**) in each group by ELISA. The data are presented as the means ± SD. **P < 0.01 vs the control group; ^##^*P* < 0.01 vs the LPS group; ^&&^*P* < 0.01 vs the LPS+Dex group

LPS induces neutrophil activation, which in turn releases inflammatory cytokines that damage alveolar epithelial cells, leading to increased pulmonary edema. We next analyzed the effect of Dex on the concentrations of TNF-α, IL-1β, IL-6, and IL-10 in bronchoalveolar lavage (BALF). Compared with those in the control group, the concentrations of TNF-α, IL-1β, IL-6, and IL-10 in BALF were increased in the LPS group, while Dex alleviated the concentrations of TNF-α (Fig. 3a), IL-1β (Fig. 3b), and IL-6 (Fig. 3c) and increased the concentration of IL-10 (Fig. 3d). However, the PI3K inhibitor LY294002 partially reversed these effects of Dex.

### Dexmedetomidine alleviated pulmonary edema and promoted alveolar fluid clearance in LPS-induced ALI

**Fig. 4:**
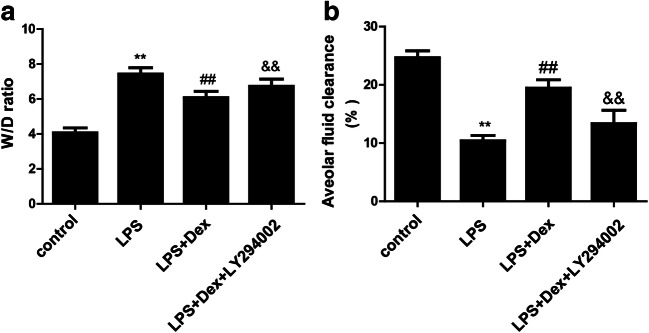
Effect of Dex on pulmonary edema in LPS-induced ALI. Rats with LPS (20 mg/kg)-induced ALI were treated with Dex (100 μg/kg). To investigate whether the protective effect of Dex is PI3K-dependent, LY294002 (3 mg/kg), a PI3K inhibitor, was given 30 min prior to LPS administration. Eight hours later, the rats were sacrificed by bloodletting. **a** Right lung tissues were harvested to measure the W/D ratio. **b** Albumin (5%) solution containing Evans blue-labeled albumin (5 ml/kg) was instilled into the left lung through a tracheostomy, and AFC was calculated. The data are presented as the means ± SD. **P < 0.01 vs the control group; ^##^*P* < 0.01 vs the LPS group; ^&&^*P* < 0.01 vs the LPS+Dex group

Pulmonary edema is a landmark event in ALI, and it is the main cause of hypoxemia. We calculated the effect of Dex on the lung W/D ratio in LPS-induced ALI. Compared with that in the control group, the W/D ratio was increased in the LPS group, while Dex treatment significantly alleviated the W/D ratio (Fig. 4a). Decreased alveolar fluid clearance (AFC) is an important mechanism that leads to pulmonary edema in ALI. We also examined the effect of Dex on AFC. Compared with that in the control group, the AFC was decreased in the LPS group, while Dex treatment increased the AFC (Fig. 4b). However, LY294002, a PI3K inhibitor, partly prevented the protective effects of Dex. These results indicated that Dex attenuates pulmonary edema in a PI3K-dependent manner.

### Dexmedetomidine improved PaO_2_ in LPS-induced ALI

**Fig. 5:**
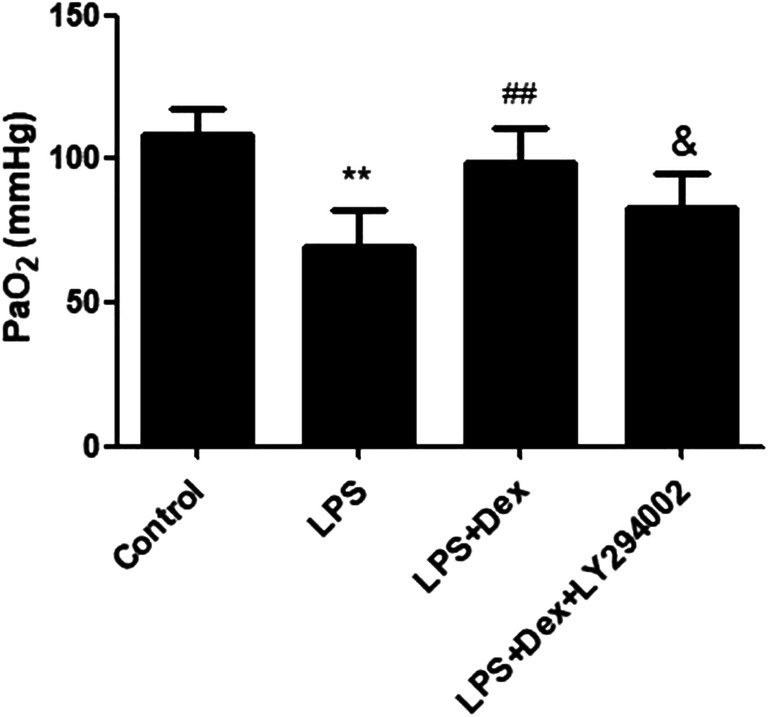
Effect of dexmedetomidine on PaO_2_. The effect of Dex on pulmonary edema in LPS-induced ALI was studied. Rats with LPS (20 mg/kg)-induced ALI were treated with Dex (100 μg/kg). To investigate whether the protective effect of Dex is PI3K-dependent, LY294002 (3 mg/kg), a PI3K inhibitor, was given 30 min prior to LPS administration. Before the rats were sacrificed, 0.5 ml blood was taken from the common carotid artery to detect PaO_2_. The data are presented as the means ± SD. **P < 0.01 vs the control group; ^##^*P* < 0.01 vs the LPS group; ^&^*P* < 0.05 vs the LPS+Dex group

Hypoxemia leads to reduced oxygenation and induces organ injury. In this experiment, we calculated the effect of Dex on PaO_2_. Compared with that in the control group, PaO_2_ was decreased in the LPS group, while Dex treatment significantly increased PaO_2_ (Fig. 5).

### Dexmedetomidine increased the expression of ENaC in LPS-induced ALI

To confirm that Dex stimulates AFC by increasing the expression of ENaC, the expression levels of α-, β-, and γ-ENaC in rat lung tissues were detected by western blotting. Furthermore, immunohistochemical analysis was employed to assess α-, β-, and γ-ENaC levels in rat lung tissues.

**Fig. 6:**
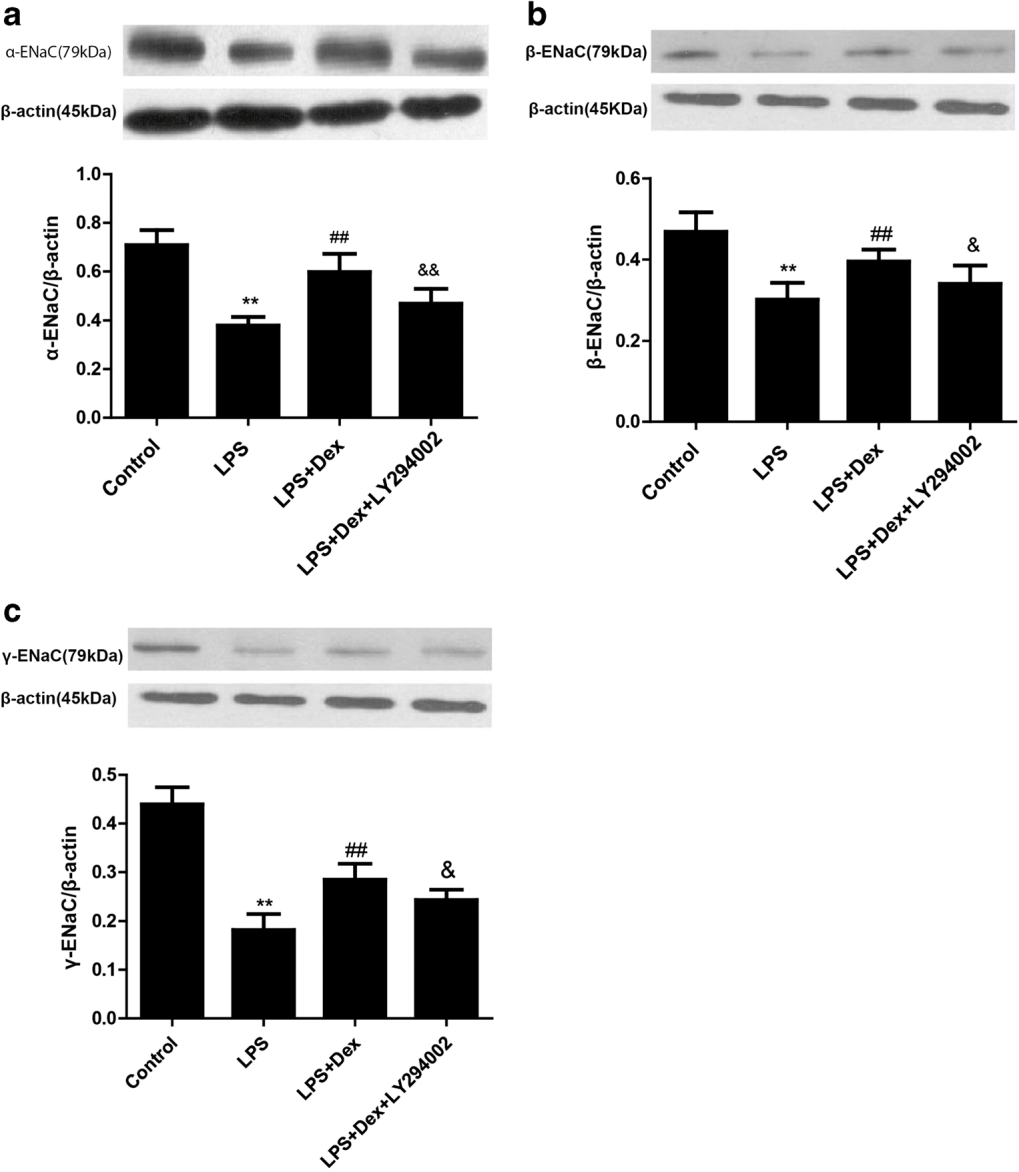
Effect of Dex on the expression of ENaC in LPS-induced ALI. Rats with LPS (20 mg/kg)-induced ALI were treated with Dex (100 μg/kg). To investigate whether the protective effect of Dex is PI3K-dependent, LY294002 (3 mg/kg), a PI3K inhibitor, was given 30 min prior to LPS administration. Eight hours later, the rats were sacrificed by bloodletting. Left lung tissues were harvested to assess the levels of α-ENaC (**a**), β-ENaC (**b**), and γ-ENaC (**c**) by western blotting. The data are presented as the means ± SD. **P < 0.01 vs the control group; ^##^*P* < 0.01 vs the LPS group; ^&&^*P* < 0.01, ^&^*P* < 0.05, vs the LPS+Dex group

Compared with those in the control group, the expression levels of α-, β-, γ-ENaC in rat lung tissues were downregulated in the LPS group, while Dex treatment increased the expression of α-ENaC (Fig. 6a), β-ENaC (Fig. 6b), and γ-ENaC (Fig. 6c). However, the PI3K inhibitor partially prevented the protective effects of Dex.

**Fig. 7:**
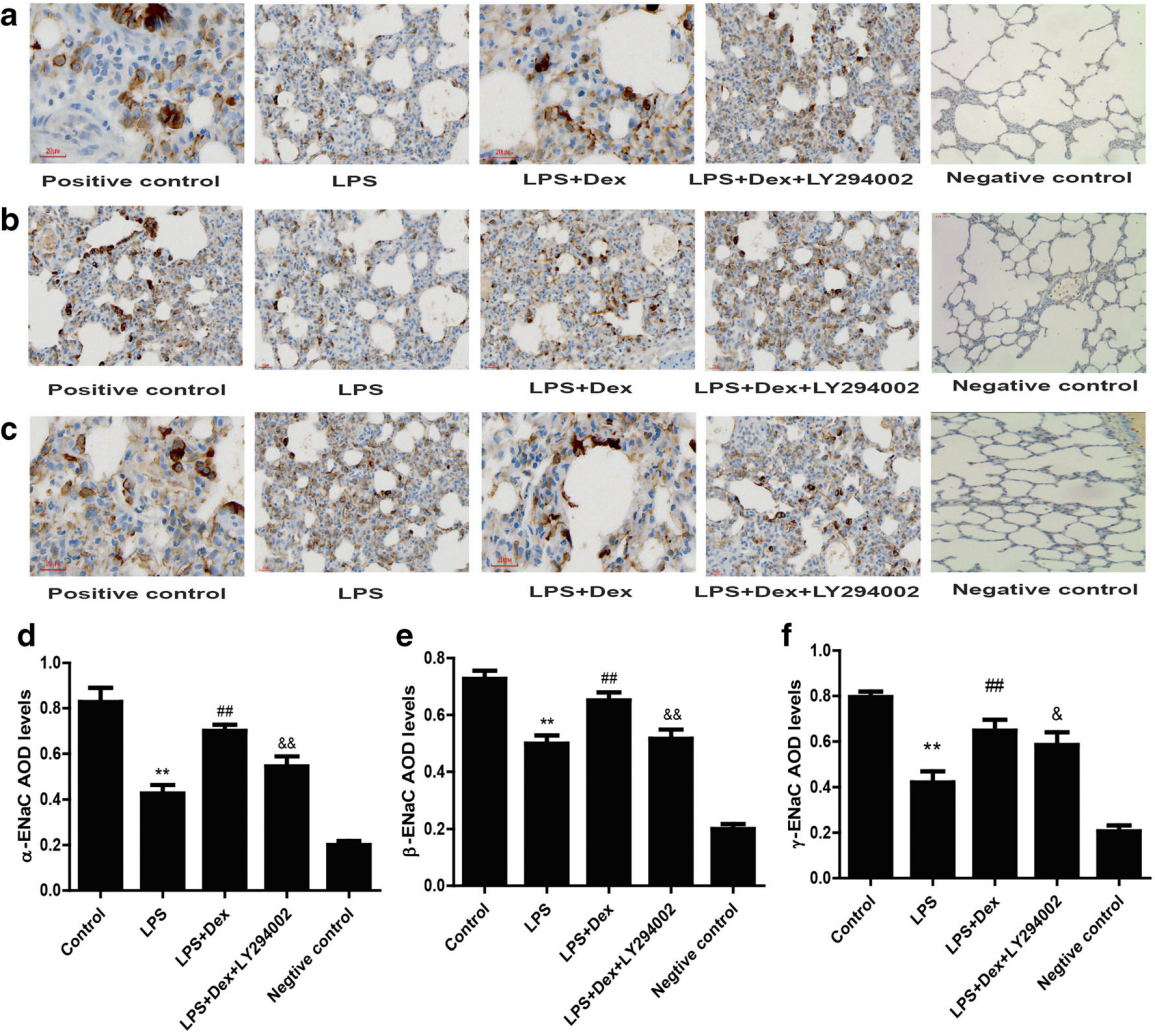
Effect of Dex on the expression of ENaC in LPS-induced ALI. Rats with LPS (20 mg/kg)-induced ALI were treated with Dex (100 μg/kg). To investigate whether the protective effect of Dex is PI3K-dependent, LY294002 (3 mg/kg), a PI3K inhibitor, was given 30 min prior to LPS administration. Eight hours later, the rats were sacrificed by bloodletting. Left lung tissues were harvested to assess the levels of α-ENaC (**a**), β-ENaC (**b**), and γ-ENaC (**c**) by immunohistochemistry. Densitometric quantification of the levels of α-ENaC (**d**), β-ENaC (**e**), and γ-ENaC (**f**). The data are presented as the means ± SD. **P < 0.01 vs the control group; ^##^*P* < 0.01 vs the LPS group; ^&&^*P* < 0.01, ^&^*P* < 0.05 vs the LPS+Dex group

Immunohistochemical analysis was used to determine the expression of α-, β-, γ-ENaC in the rat lungs. Immunostained cells appeared brown. The expression of α-, β-, and γ-ENaC was decreased in the LPS group compared with the control group but increased in the LPS+Dex group compared with the LPS group (Fig 7a–f). However, the PI3K inhibitor partially prevented the protective effects of Dex. These results indicate that Dex acts through PI3K to increase ENaC expression.

### Dose- and time-dependent regulation of ENaC expression by LPS in A549 cells

**Fig. 8:**
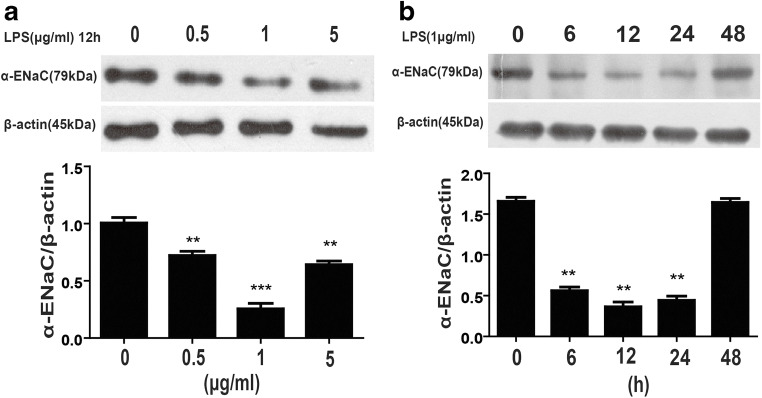
Effect of LPS on α-ENaC expression in vitro. A549 cells were stimulated with LPS at different concentrations for 12 h, and the expression of α-ENaC (**a**) was detected by western blotting. A549 cells were stimulated with 1 μg/ml LPS, and western blotting was used to detect the expression of α-ENaC (**b**) at different times. The data are presented as the means ± SD. **P < 0.01 vs the control group; ***P < 0.001 vs the control group

To determine the relationship between the dose and stimulation time of LPS and the expression of ENaC, we first stimulated A549 cells with different concentrations of LPS (0, 0.5, 1, 5 μg/ml) and detected the expression of α-ENaC by western blotting. The results showed that the expression of α-ENaC was dose-dependent and that the dose of 1 μg/ml produced the most significant effect. Accordingly, 1 μg/ml LPS was used in subsequent cell experiments (Fig. 8a)

To determine the expression of α-ENaC in A549 cells at different times, cells were stimulated with 1 μg/ml LPS for 0, 6, 12, 24, or 48 h. The results showed that the expression of α-ENaC was decreased at 6, 12, and 24 h and that the decrease was the most obvious at 12 h. Therefore, in the subsequent cell experiments, the LPS stimulation time was 12 h (Fig. 8b)

### Dexmedetomidine increased cell viability and decreased LDH activity in LPS-stimulated A549 cells

**Fig. 9:**
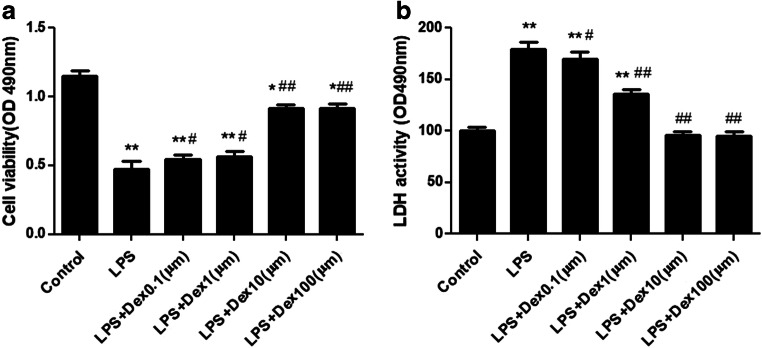
**a** Effect of Dex on cell viability and LDH activity in vitro. A549 cells were stimulated by LPS and then treated with different concentrations of Dex (0.1, 1, 10, or 100 μm) for 12 h. **b** Effects of different concentrations of Dex on LDH activity in A549 cells treated with LPS for 12 h. Cell viability and LDH activity were detected. The data are presented as the means ± SD. *P < 0.05, **P < 0.01 vs the control group; ^#^*P* < 0.05, ^##^*P* < 0.01 vs the control group

We evaluated the effects of Dex on cell viability and LDH activity. A549 cells were treated with Dex at different concentrations (0.1, 1, 10, or 100 μM) in the presence of LPS (1 μg/ml) for 12 h. The results showed that both 10 μm and 100 μm Dex effectively increased cell activity (Fig. 9a) and decreased LDH activity (Fig. 9b) in the presence of LPS. Therefore, Dex (10 μm) was used in subsequent cell experiments.

### Dexmedetomidine upregulated the expression of ENaC in LPS-stimulated A549 cells

**Fig. 10:**
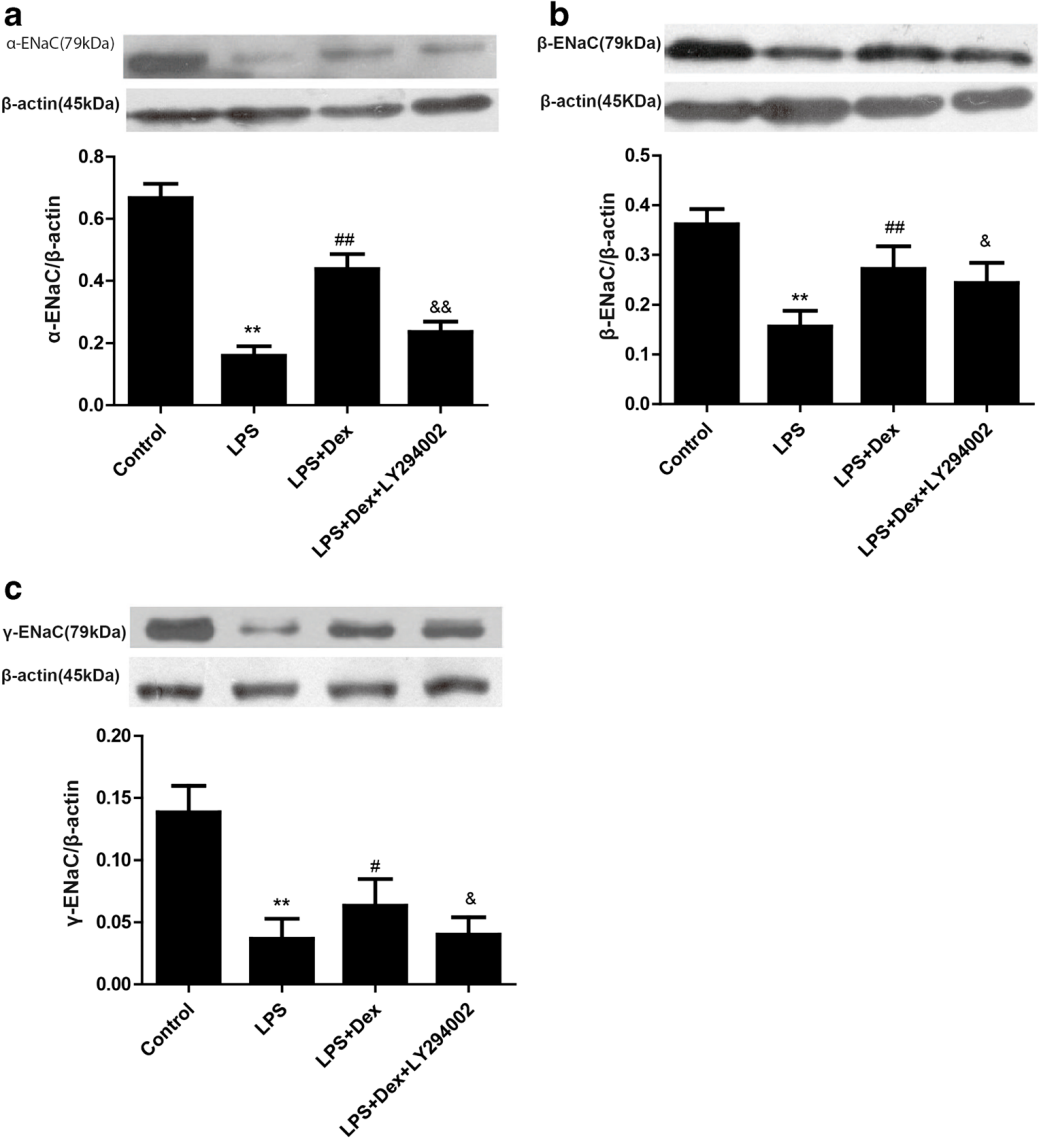
Effect of Dex on the expression of ENaC in vitro. A549 cells were treated with LY294002 (10 μΜ) 30 min prior to LPS (1 μg/ml) administration and were then treated with Dex (10 μΜ). The protein expression of α-ENaC (**a**), β-ENaC (**b**), and γ-ENaC (**c**) was assessed by western blotting 12 h after LPS treatment. The data are presented as the means ± SD. **P < 0.01 vs the control group; ^#^*P* < 0.05, ^##^*P* < 0.01 vs the LPS group; ^&^*P* < 0.05, ^&&^*P* < 0.01 vs the LPS+Dex group

To further confirm that Dex stimulates AFC by increasing the expression of ENaC, we detected the expression levels of α-, β-, and γ-ENaC in A549 cells. Compared with those in the control group, the expression levels of α-, β-, and γ-ENaC were downregulated in A549 cells in the LPS group, while Dex increased the expression of α-ENaC (Fig. 10a), β-ENaC (Fig. 10b), and γ-ENaC (Fig. 10c). However, the PI3K inhibitor partially prevented the effects of Dex.

### PI3K activates the Akt/Nedd4-2 pathway in LPS-stimulated A549 cells

**Fig. 11:**
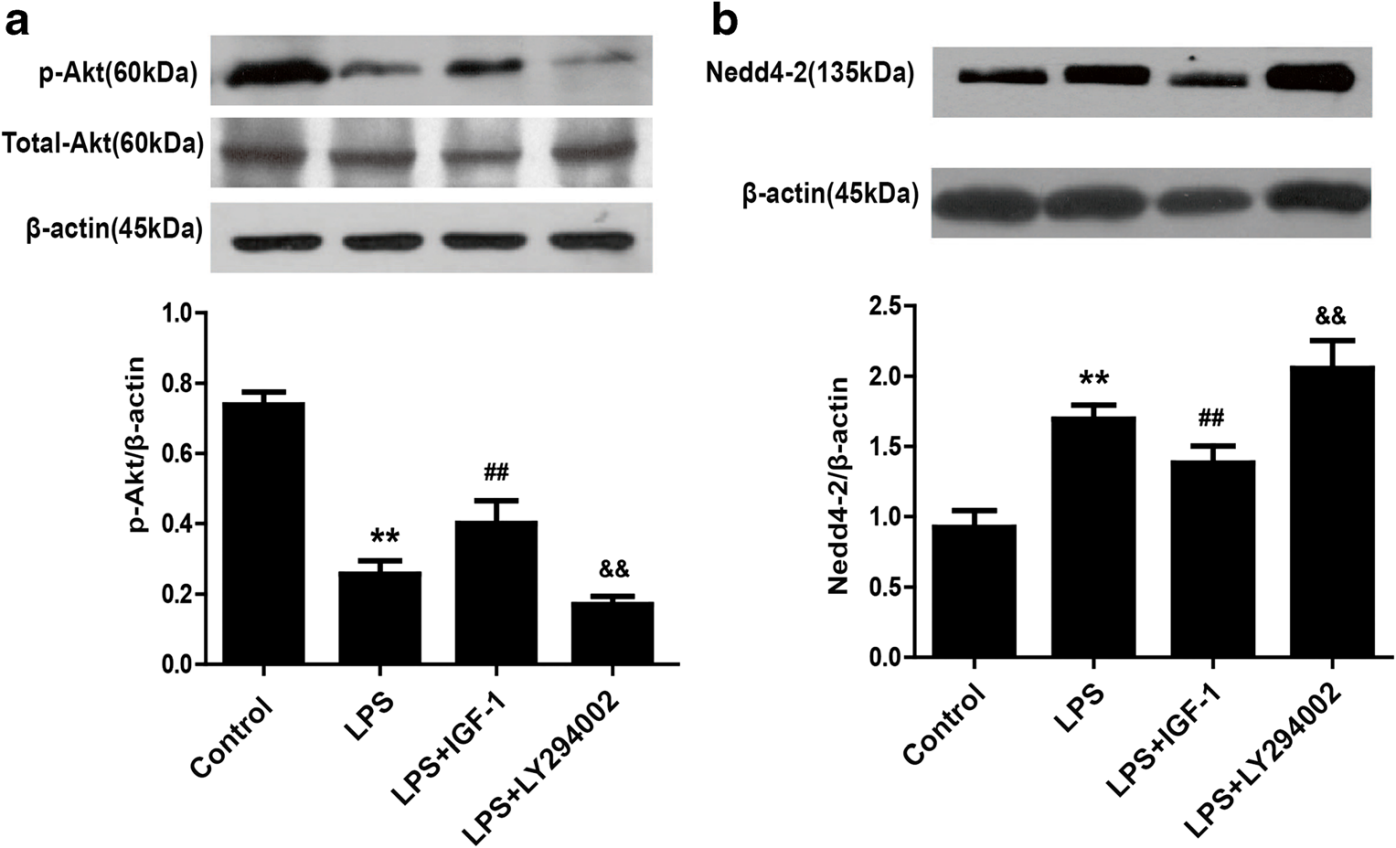
Effect of PI3K on the expression of phosphorylated Akt and Nedd4-2 in LPS-stimulated A549 cells. A549 cells were treated with LY294002 (10 μΜ) 30 min prior to LPS (1 μg/ml) administration and IGF-1 (200 ng/ml) 1 h prior to LPS (1 μg/ml) administration. The protein expression of p-Akt (**a**) and Nedd4-2 (**b**) was assessed by western blotting 12 h after LPS treatment. The data are presented as the means ± SD. **P < 0.01 vs the control group; ^##^*P* < 0.01, ^&&^*P* < 0.01 vs the LPS group

To investigate whether Akt and Nedd4-2 are downstream of PI3K, we administered PI3K agonists and inhibitors respectively in LPS-stimulated A549 cell to observe the expression of Akt and Nedd4-2. Compared with that in the control group, the protein level of phosphorylated Akt (p-Akt) was decreased and Nedd4-2 was increased in the LPS group, while IGF-1 increased phosphorylated Akt expression and decreased Nedd4-2 expression. However, the LY294002 decreased phosphorylated Akt expression and increased Nedd4-2 expression. These results indicate that P-Akt and Nedd4-2 are downstream of PI3K (Fig. 11a, b).

### Dexmedetomidine activated the PI3K/Akt/Nedd4-2 signaling pathway in vivo and in vitro

**Fig. 12:**
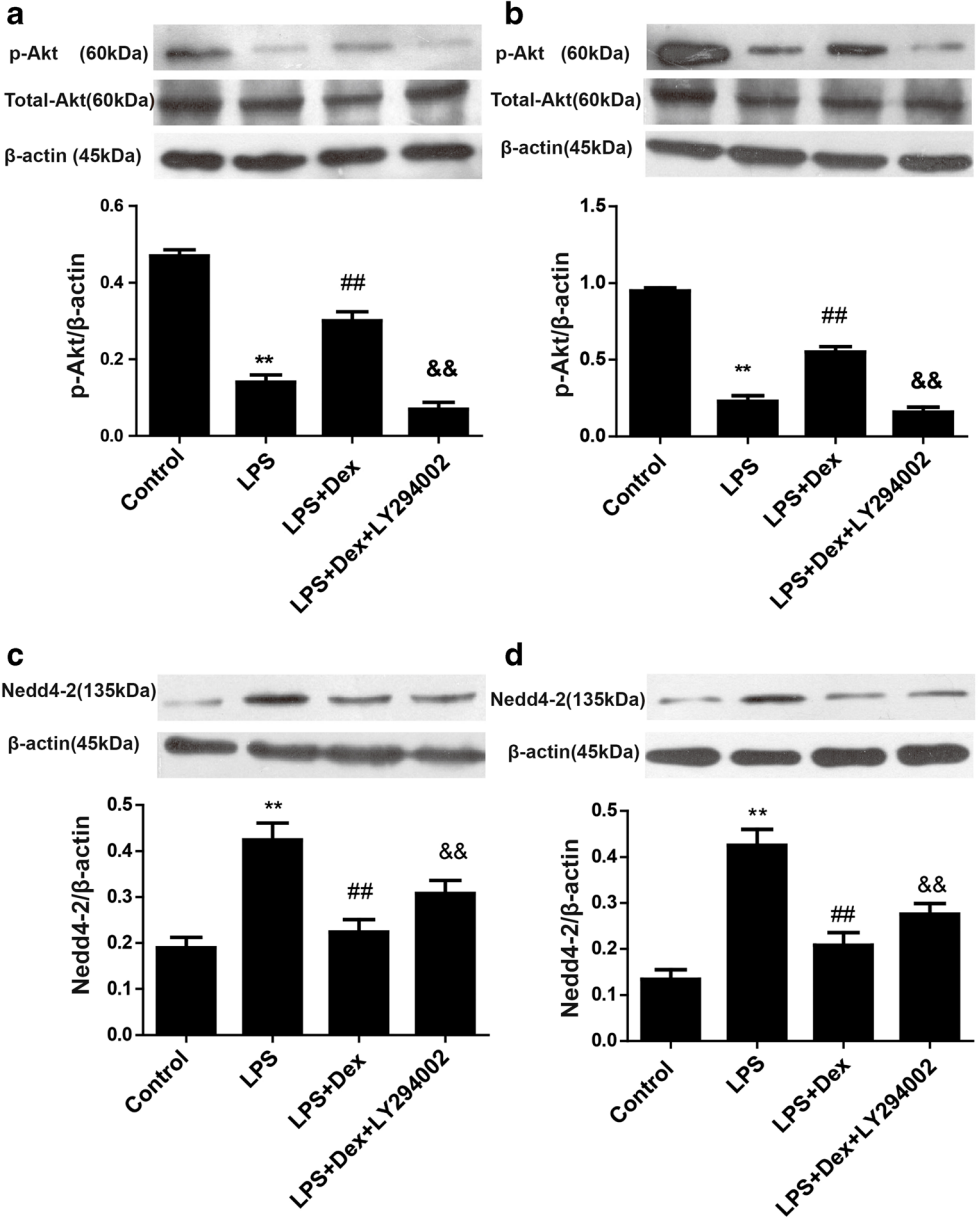
Effect of Dex on the expression of phosphorylated Akt and Nedd4-2 in vivo and in vitro. A549 cells were treated with LY294002 (10 μΜ) 30 min prior to LPS (1 μg/ml) administration and were then treated with Dex (10 μΜ). Western blotting was used to assess the levels of phosphorylated Akt in LPS-induced ALI (**a**) and LPS-stimulated A549 cells (**b**). Western blotting was used to assess the levels of Nedd4-2 in LPS-induced ALI (**c**) and LPS-stimulated A549 cells (**d**). The data are presented as the means ± SD. **P < 0.01 vs the control group; ^##^*P* < 0.01 vs the LPS group; ^&&^*P* < 0.05 vs the LPS+Dex group

As the effect of Dex on ENaC expression appeared to be PI3K dependent and PI3K activates the Akt/Nedd4-2 pathway, here we investigated whether Dex regulates the expression of ENaC through the PI3K/Akt/Nedd4-2 signaling pathway, phosphorylated Akt, and Nedd4-2 were measured by western blotting. Compared with that in the control group, the protein level of phosphorylated Akt was decreased in the LPS group in vivo and in vitro, while Dex increased phosphorylated Akt expression (Fig. 12a, b). Compared with that in the control group, the protein level of Nedd4-2 was increased in the LPS group in vivo and vitro, while Dex decreased Nedd4-2 expression (Fig. 12c, d). However, the PI3K inhibitor LY294002 reversed these effects of Dex, indicating that Dex activates the PI3K/Akt/Nedd4-2 signaling pathway.

## Discussion

Our study showed that LPS induced lung tissue injury, which was characterized by increased neutrophil infiltration in lung tissue, alveolar structure destruction, and interstitial edema. Dex treatment reduced lung histopathological injury in rats. Hypoxemia is primarily caused by pulmonary edema in patients with ALI. Our data suggested that Dex alleviated pulmonary edema and improved hypoxemia in LPS-induced ALI. Moreover, impaired AFC was observed in the LPS group, and Dex increased AFC. These data indicated that Dex alleviated LPS-induced ALI through alleviating pulmonary edema and improving hypoxemia. In the current study, we also confirmed that LY294002, a PI3K inhibitor, reversed the protective effect of Dex, suggesting that these effects of Dex were PI3K-dependent. Our previous studies showed that Dex reduces pulmonary edema by increasing aquaporin (AQP) expression in LPS-induced ALI [17]. However, ENaC is thought to be the rate-limiting factor for the reabsorption of pulmonary edema. ENaC is mainly composed of three homologous subunits (α-, β-, and γ-ENaC), which are expressed in alveolar epithelial cells. The α subunit is necessary in the transport of sodium ions, while the β and γ subunits promote channel activity. Recent studies have revealed that upregulated ENaC expression promotes AFC and reduces pulmonary edema in ALI animal models [22]. Clinical trials have also shown that inhalation of an ENaC activator significantly reduces the extravascular lung water index (EVLWI) in ARDS patients [23]. In this experiment, we observed that ENaC expression was low in the LPS group, while Dex increased α-, β-, and γ-ENaC expression in LPS-induced ALI and LPS-stimulated A549 cells. However, the PI3K inhibitor LY294002 blocked these effects of Dex. In summary, our results suggest that Dex reduces pulmonary edema through stimulating AFC by increasing the expression of ENaC and that this effect is PI3K-dependent.

Studies have shown that the expression of the inflammatory cytokines TNF-α and IL-1β are increased significantly in ALI [24, 25]. It has been reported that Dex can restrain lung tissue inflammatory response in various pathological settings such as sepsis, hemorrhagic shock, and ischemia-reperfusion [26–28]; our previous studies also confirmed that TNF-α and IL-1β concentrations in lung tissues are significantly increased in LPS-induced ALI models, while Dex reduces TNF-α and IL-1β concentrations [17]. Consistent with these studies, our study revealed that Dex reduced TNF-α, IL-1β, and IL-6 concentrations and increased IL-10 concentrations in BALF, suggesting that Dex may be beneficial for regulating the balance of inflammatory responses. In the present study, these beneficial effects of Dex were abrogated by a PI3K inhibitor (LY294002), indicating that the anti-inflammatory effect of Dex is PI3K-dependent. These results are consistent with previous studies demonstrating that PI3K/Akt pathway plays a crucial role in the attenuation of inflammation [29, 30]. At present, the exact anti-inflammatory mechanism of Dex is not clear. Some studies have shown that Dex has an anti-inflammatory effects by activating α_2_-adrenoceptor (α_2_AR)[31–33]. In accordance with these studies, our previous studies have reported that Dex alleviates the inflammatory response induced by hemorrhagic shock/resuscitation-endotoxemia, while the administration of an α_2_AR inhibitor reverses the anti-inflammatory effects of Dex [13], suggesting α_2_AR-mediated effect is an important mechanism. However, other studies have demonstrated that Dex mitigated inflammatory response through other receptors, such as cannabinoid receptor2 (CB_2_), α_7_ nicotinic acetylcholine receptors (α_7_nAChR) [34, 35]. Additionally, investigators found that Dex plays an anti-inflammatory role by activating the α_2_AR /PI3K/Akt signaling pathway and then upregulating the cholinergic anti-inflammatory pathway in a rat model of spinal cord injury [36]. So, we hypothesized that Dex inhibits inflammatory responses by activating α_2_AR, followed by upregulating the PI3K/Akt signaling pathway. In this study, although we confirmed the Dex has an anti-inflammatory effect and is PI3K-dependent, whether it is related to α_2_AR is still elucidated.

In this study, we primarily ascertained the effect of Dex on the reduction of pulmonary edema from the perspective of alveolar fluid clearance. Our study have found that Dex alleviated pulmonary edema through stimulating alveolar fluid clearance by upregulating the expression of ENaC. Study demonstrated that some proinflammatory cytokines such as TNF-α and IL-1β inhibit fluid transport in distal lung epithelium through reducing the expression of ENaC [10, 37]. Given the anti-inflammatory effects of Dex by downregulating proinflammatory cytokines such as TNF-α, IL-1β, and IL-6, and upregulating anti-inflammatory cytokines such as IL-10, it is possible that Dex increases the expression of ENaC by preventing excessive inflammatory responses in LPS-induced ALI. However, some proinflammatory cytokines such as TNF-α and IL-1β not only inhibit fluid transport but also increase alveolar permeability through disrupting tight junction barriers and endothelial integrity [37]. Thus, in the present study, it is likely that the effect of Dex in reducing pulmonary edema may be related to the reduction of endothelial leakage and permeability.

Our study also revealed that Dex plays a protective role by reducing pulmonary edema through increasing ENaC-mediated AFC. However, the mechanism is not clear. In recent years, preliminary studies have been conducted on the relevant signaling pathways that can cause changes in ENaC expression, including the glucocorticoid receptor signaling pathway [38] and the β_2_-adrenergic receptor agonist-mediated CAMP-PKA signaling pathway [39, 40], but these pathways are not ideal for clinical treatment, and their involvement remains controversial. The lipid kinase PI3K generates phosphatidylinositol (3,4,5)-trisphosphate (PIP3), which is a second messenger that facilitates the translocation of AKT to the plasma membrane. At the membrane, Akt is phosphorylated and plays an important role in processes such as cell proliferation, differentiation, survival, and apoptosis. Previous studies have shown that the activation of the PI3K/Akt pathway decreases pulmonary edema by upregulating the expression of ENaC in LPS-induced ALI [20, 41]. Experimental studies have revealed that stimulation with LPS reduces the expression of p-Akt [42]. Our study showed that Dex inhibited the reduction in p-Akt expression in LPS-induced ALI and LPS-stimulated A549 cells. However, a PI3K inhibitor (LY294002) reversed the effect of Dex, suggesting that Dex acts through PI3K-dependent action of Akt.

Nedd4-2 is a ubiquitin-protein ligase that mediates ubiquitination and degradation of ion channel proteins. The carboxyl terminals of the α, β, and γ subunits of ENaC have PY modules, and the ww domain of Nedd4-2 is bound to the PY modules of ENaC to promote ubiquitination of ENaC and reduce the expression of ENaC on the cell membrane. Recent studies have shown that degradation of ENaC mediated by ubiquitination of Nedd4-2 is involved in the occurrence and development of acute pulmonary edema [43]. In addition, the Akt/Nedd4-2 pathway plays an important role in regulating ENaC [44]. Akt attenuates ENaC ubiquitination by phosphorylating Nedd4-2, thereby increasing ENaC expression. In this study, we found that Dex decreased the expression of Nedd4-2. However, the beneficial effects of Dex were inhibited by LY294002 in vivo and in vitro, suggesting that PI3K-dependent activation of Nedd4-2 may be involved in the upregulation of ENaC expression. To further investigate whether Akt and NEDD4-2 are downstream of PI3K, additional cell experiments were performed. Our study showed that a PI3K agonists (IGF-1) markedly promoted the p-Akt expression and decreased the Nedd4-2 expression, while PI3K inhibitor LY294002 decreased the expression of p-Akt and increased the Nedd4-2 expression. The above results indicated that Dex promoted ENaC expression by activating PI3K/Akt/ Nedd4-2 signaling pathway

In summary, our study demonstrates that Dex alleviates pulmonary edema not only by inhibiting inflammation but also by the upregulation of ENaC expression in LPS-induced ALI. This protective effect is attributed to the action of the PI3K/Akt/Nedd4-2 signaling pathway. Our results provide new insight into alleviating pulmonary edema in LPS-induced ALI and suggest a new therapeutic use for Dex in patients with ALI. However, this study only provides some limited data to indicate that Dex may reduce pulmonary edema through stimulating ENaC expression in LPS-induced ALI in rats. In addition, DEX was prophylactically used, and the doses were different from that used in human. So, the clinical significance of its usage to alleviate pulmonary edema in patients with ALI/ARDS should be further evaluated.
